# Editorial: Novel Insights Into Ferroptosis

**DOI:** 10.3389/fcell.2021.754160

**Published:** 2021-10-07

**Authors:** Yasaman Setayeshpour, Jen-Tsan Chi

**Affiliations:** ^1^Department of Molecular Genetics and Microbiology, Duke University Medical Center, Durham, NC, United States; ^2^Duke Center for Genomic and Computational Biology, Durham, NC, United States

**Keywords:** ferroptosis, metastasis, YAP, TAZ, DDR2, serum, lymph, GPX4

Most cancer deaths are caused by metastatic cancers (Hanahan and Weinberg, 2000; Gupta and Massagué, 2006; Seyfried and Huysentruyt, 2013). Consequently, better therapeutic approaches are urgently needed for targeting metastatic cancer cells. Recently, ferroptosis, a newly recognized form of programmed cell death, is being acknowledged as an important tumor suppression mechanism with a significant therapeutic potential (Lei et al., 2021). In this editorial, we review recent evidence that suggest ferroptosis may be especially relevant as the metabolic Achilles' heel of metastatic cancer cells. We further review how triggering ferroptosis may hold a significant therapeutic potential for preventing and treating metastatic cancer.

Ferroptosis is a recently appreciated cell death as coined by Dixon et al., but it is an ancient form of programed cell death that is mechanistically and morphologically distinct from apoptosis, autophagy, and necrosis. Ferroptosis is usually triggered by oxidative stress and characterized by lipid peroxide accumulation and iron imbalance in the cell. The depletion of the antioxidant glutathione (GSH) and the loss of activity from the lipid repair enzyme glutathione peroxidase 4 (GPX4) prevents the metabolism of lipid peroxides, which in turn leads to Fe^2+^ oxidization of lipids and the massive accumulation of lipid reactive oxygen species (ROS) (Dixon et al., 2012; Li et al., 2020; Jiang et al., 2021). Additional ferroptosis protection mechanisms include ferroptosis suppressor protein 1 (FSP1) (Bersuker et al., 2019; Doll et al., 2019) and dihydroorotate dehydrogenase (DHODH) (Mao et al., 2021), which generate ubiquinol to defend against ferroptosis on the plasma membrane and inner mitochondrial membrane, respectively. This unique type of programmed cell death has been recognized as a major tumor suppression mechanism and possesses therapeutic potential for eradicating tumor cells (Lei et al., 2021). Experimental drugs, such as erastin, which inhibits the cystine import system ${\text{x}}_{\text{c}}^{-}$, and RSL3 and FIN56, which inhibit GPX4, cystine deprivation, and knockdown of established anti-ferroptotic genes have shown cancer cells to be sensitive to ferroptosis-inducing treatments (Li et al., 2020).

Most intriguingly, many metastasis-associated processes are found to promote ferroptosis (Brown et al., 2017; Viswanathan et al., 2017). Metastasis transpires in multiple steps of a metastatic cascade. These processes start with the local infiltration of the tumor cells into adjacent tissues (Hapach et al., 2019). This first step involves the epithelial-mesenchymal transition (EMT) of cancer cells that prompt epithelial cells to assume “mesenchymal” migratory and invasive phenotypes. EMT is strongly associated with metastasis (Mittal, 2018). Interestingly, several EMT regulators are recognized to promote ferroptosis and cystine addictions (Tang et al., 2017; Viswanathan et al., 2017). Following EMT, cancer cells detach from the extracellular matrix (ECM) and adjacent cells, which enable the migration and dissemination of tumor cells (Brown et al., 2017; Hapach et al., 2019). Wu et al. (2019) show that cell-cell contact can protect against ferroptosis and hence tumor cells going through EMT and ECM detachment are especially vulnerable to ferroptosis. We have also reported that low cell density and loss of cell-cell contact promote ferroptosis through the activation of YAP (Yes-associated protein 1) (Yang et al., 2021) or TAZ (transcriptional coactivator with PDZ-binding motif) (Yang et al., 2019, 2020); we showed that TAZ activation enhances ferroptosis at low cell density and *TAZ* removal renders cells resistant to ferroptosis. Importantly, both YAP and TAZ are critical regulators of multiple steps in the metastatic cascade (Piccolo et al., 2014; Zanconato et al., 2016). Wu et al. (2019) further establish this finding by showing that high cell density enhances cellular contacts and suppresses ferroptosis through inhibiting YAP. YAP/TAZ are critical for tumor development and its metastasis via the regulation and transduction of the cellular structure in the tumor microenvironment (Zanconato et al., 2016). Another metastasis-promoting factor is the increased stromal collagen deposition through the collagen receptor discoidin domain receptor 2 (DDR2) (Zhang et al., 2013; Gonzalez et al., 2017). DDR2 is concordantly upregulated in metastatic cancer and maintains a fibroblastic phenotype. Interestingly, we report in Lin et al. (2021) that DDR2 is essential for ferroptosis susceptibility. Given the ability of YAP/TAZ and DDR2 to promote both ferroptosis and metastasis, triggering ferroptosis is an incredibly promising approach to target cancer cells at their initial stage of metastasis.

The metastatic process also involves the intravasation of tumor cells into the vasculature or lymphatic system, in which the cells must survive while traveling in the circulatory systems before extravasation from the circulation and colonization of a secondary tumor site (Hapach et al., 2019). At this stage, too, tumor cells are particularly susceptible to ferroptosis, but Ubellacker et al. (2020) shows that metastatic melanoma cells undergo significant ferroptosis in the blood stream and only survive in the lymphatic system when protected from ferroptosis by lymph. In further support of this finding, Magtanong et al. (2019) also report that extrinsic monosaturated fatty acids (MUFAs) protect cancer cells against ferroptosis by blocking the accumulation of lipid peroxides in the plasma membrane in an A synthetase long-chain family member 3 (ACSL3)-dependent manner. Yet another study, Hong et al. (2021), found that the circulating cancer cells (CTC) employ the lipogenesis regulator, SREBP2 (Sterol Regulatory Element Binding Transcription Factor 2) to resist ferroptosis by the induction of transferrin, which reduces intracellular iron pools. Collectively, these studies point to the major influence of the intrinsic and extrinsic factors that enable CTC to survive ferroptosis, which further highlights the significant potential of targeting such protections for treatment of metastasizing cancer cells.

In summary, evidence is accumulating to suggest that ferroptosis shows an incredibly promising potential as a therapeutic approach for metastatic tumors. Several agents have been developed to trigger ferroptosis *in vivo* to further characterize and understand the therapeutic potential of ferroptosis. For example, Imidazole ketone erastin (IKE), an erastin analog, and the human engineered Cyst(e)inase (Cramer et al., 2017) can be used to trigger *in vivo* ferroptosis. Cyst(e)inase can also synergize with immunotherapy (Wang et al., 2019) and is effective in pancreatic cancers (Badgley et al., 2020). Therefore, these reagents will need to be further optimized for the future clinical application in patients. However, the use of ferroptosis as a therapeutics approach in the setting of metastatic cancers is still in development. Understanding what mechanisms to disable and/or how to manipulate the microenvironment of tumor cells so they become sensitive to ferroptosis would have a significant impact in the field of cancer genetics and will accelerate the progress and possibility of using ferroptosis in novel therapeutic approaches for millions of patients with metastatic cancer.

## Author Contributions

YS and J-TC wrote the manuscript together. All authors contributed to the article and approved the submitted version.

## Funding

We acknowledge the financial support in part by DoD grants (W81XWH-17-1-0143, W81XWH-15-1-0486, W81XWH-19-1-0842, and W81XWH-20-1-0907) and NIH grants (1R21-AI149205).

## Conflict of Interest

The authors declare that the research was conducted in the absence of any commercial or financial relationships that could be construed as a potential conflict of interest.

## Publisher's Note

All claims expressed in this article are solely those of the authors and do not necessarily represent those of their affiliated organizations, or those of the publisher, the editors and the reviewers. Any product that may be evaluated in this article, or claim that may be made by its manufacturer, is not guaranteed or endorsed by the publisher.
